# RNA-Seq Analysis Implicates Detoxification Pathways in Ovine Mycotoxin Resistance

**DOI:** 10.1371/journal.pone.0099975

**Published:** 2014-06-17

**Authors:** Jinbi Zhang, Zengxiang Pan, Stephanie Moloney, Allan Sheppard

**Affiliations:** Liggins Institute, University of Auckland, Auckland, New Zealand; University Paris South, France

## Abstract

Mycotoxin induced hepatoxocity has been linked to oxidative stress, resulting from either an increase in levels of reactive oxygen species (ROS) above normal levels and/or the suppression of antioxidant protective pathways. However, few detailed molecular studies of mycotoxicoses in animals have been carried out. This study use current RNA-seq based approaches to investigate the effects of mycotoxin exposure in a ruminant model. Having first assembled a de novo reference transcriptome, we use RNA-Seq technology to define *in vivo* hepatic gene expression changes resulting from mycotoxin exposure in relationship to pathological effect. As expected, characteristic oxidative stress related gene expression is markedly different in animals exhibiting poorer outcomes. However, expression of multiple genes critical for detoxification, particularly members of the cytochrome P450 gene family, was significantly higher in animals exhibiting mycotoxin tolerance (‘resistance’). Further, we present novel evidence for the amplification of Wnt signalling pathway activity in ‘resistant’ animals, resulting from the marked suppression of multiple key Wnt inhibitor genes. Notably, ‘resistance’ may be determined primarily by the ability of an individual to *detoxify* secondary metabolites generated by the metabolism of mycotoxins and the potentiation of Wnt signalling may be pivotal to achieving a favourable outcome upon challenge.

## Introduction

Mycotoxicoses resulting from the inhalation or ingestion of microfungal spores occurs commonly across the world [1] and increasingly, mycotoxins are having an impact on global food ‘security’ [2]. Specifically, they represent a hazard in the agricultural sector affecting the food supply chain of both human and animals, particularly in areas where pasture and crops are impacted by challenging growing conditions and/or suboptimal food storage and handling practises [3]. Already significant, these impacts are only predicted to increase further as a consequence of global climate change [4]. In mammals, the cellular pathophysiology of many mycotoxins has often been associated cellular damage caused by oxidative stress, a result of reactive oxygen species (ROS) increasing to levels above normal and/or the suppression of antioxidant mechanisms, leading to the oxidation of DNA, protein and lipid. However, the production of damaging superoxide and hydroxyl free radicals is *not* a universal consequence of mycotoxin poisoning; indeed, the variable degree to which ROS are generated has be used as a means to classify the approximately 350 known mycotoxins as non-, moderate- or highly-oxidant [5]. Thus, while the pathological mechanisms underpinning the effects of non-oxidant mycotoxin exposure remain poorly defined, it is clear that not all cases of mycotoxicities would trigger the same enzymatic antioxidant defence mechanisms. However, as a class of xenobiotic all mycotoxin exposures would trigger detoxification mechanisms that have evolved to modify the primary toxin, thereby nullifying damaging effects by hastening their removal from the body.

A wide variety of mycotoxins are known to contaminate animal forage, and thus to have a significant economic impact on production in the agriculture sector [1]. If exposure is prolonged and/or at high enough levels, the resulting pathology develops ‘clinical’ features, and almost invariably is fatal. The economic impact of mycotoxicoses is however further exacerbated by chronic exposure to ‘sub-clinical’ levels of toxin, resulting in a marked reduction in food intake and feed conversion efficiencies. Consequentially, animals suffer ill-thrift, failing to gain weight, becoming more susceptible to disease and the loss of lactation and reproduction capacity [6] [7] [8] [9], underpinning considerable economic losses [10]. As a pastoral based economy, the New Zealand agricultural sector is notably impacted by the mycotoxin spordisemin produced by ryegrass endophyte. At times of high spore counts (under summer stress) exposure to sporidesmin is overtly hepatotoxic, a consequence of hydroxyl radical mediated oxidative damage, progressing to secondary photosensitization of skin regions not protected by fibre or fur, and the characteristic and often fatal ‘facial excema’ (FE) pathological state [11]. Meanwhile, chronic exposure to lower spore levels and the concomitant multi-seasonal accumulation of damage also results in the typical animal production losses, which associate with mycotoxin poisoning. To what extent oxidative stress damage contributes to these longer lasting effects on animal production traits is however yet to be determined.

Only a limited number of studies have utilized transcriptomics based approaches to investigate the (1) molecular consequences of xenobiotic challenges [12] [13], (2) mycotoxin exposure in agricultural animals specifically [14] and in particular, (3) to the identification of genes which impart tolerance (‘resistance’) to exposure in organisms [15]. In the present study we have used current RNA-Seq technology to explore transcriptomic based differences and inform molecular pathways which may underpin phenotypic outcomes and define exposure resistance, in a ruminant-based model of sporidesmin mycotoxin exposure. Whilst primarily of agricultural interest, our findings do suggest fundamental and novel mechanisms by which tolerance to xenobiotic challenge is mediated and ‘resistance’ thus manifest. Notably, the potential role and significance of Wnt signalling in the regulation of detoxification gene expression is highlighted.

## Materials and Methods

### Sheep Trial and Tissue Collection

The whole animal study were approved by the Ruakura Animal Ethics Committee established under the Animal Protection Regulations Act (1987, New Zealand). A total of 27 Romney cross bred sheep were challenged, in two independent ‘trials’ (n = 10 and n = 17 animals respectively) by a single oral administration of purified sporidesmin (water solution, Agresearch, NZ) at a dose rate of 0.25 mg/kg live weight [16]. Blood samples collected weekly were assessed for serum gamma-glutamy-transferase (GGT) level, as a monitor of toxin exposure and indicator of liver damage. Seven weeks after the challenge the animals were sacrificed according to commercial best practise, tissues recovered and to ‘standardize’ collection, sections of liver parenchyma approximately 20×7 mm×depth excised in each case from the left lobe, close to the division between right and left hepatic lobes. From this excision, 5×5×5 mm sections of tissue were manual cut and stored in cryotubes for immediate freezing in liquid nitrogen.

### RNA Isolation, Purification and Library Preparation

RNA isolation from snap frozen liver tissue was optimised as follows. Approximately 50–100 mg tissue was homogenized in 1 ml Trizol (Invitrogen) using a Qiagen TissueLyser II and processed using a standard Trizol protocol. Briefly, RNA was DNase treated using an Invitrogen ‘Purelink Kit’ and the quality of total RNA recovered quantified using the RNA integrity number (RIN) generated by Agilent Bioanalyzer 2100 analysis using the ‘RNA 6000 Nano-Chip’ kit (Agilent Technologies). All 26 samples with RIN>6.5 were used for downstream RT-PCR assay. Eight samples with RIN>7.5 were further processed for RNA-Seq analysis. For transcriptomic comparisons among resistant (n = 2), subclinical (n = 3) and clinical (n = 3) groups, a sequencing library was developed according to the protocol of the paired-end sample Preparation kit (Illumina, USA). Briefly, sample mRNA was enriched by using oligo(dT) magnetic beads and mRNA cleaved short fragments (about 200 bp) by adding fragmentation buffer. The first strand cDNA was synthesized by random hexamer-primer using the mRNA fragments as templates, and the second strand cDNA was synthesized by adding buffer dNTPs, RNaseH and DNA polymerase I. The short double-stranded cDNA was then purified with QiaQuick PCR extraction kit (Qiagen) and resolved with EB buffer for end repair and single nucleotide A (adenine) tail addition, and then sequencing adaptors were ligated to the fragments. The required fragments were purified by agarose gel electrophoresis and enriched by PCR amplification. At the same time, an independent pooled sequencing library was prepared following the same protocol as above, to generate a sheep transcriptome de novo assembly.

### Illumina Sequencing and Sequences Quality Control

**Figure 1:**
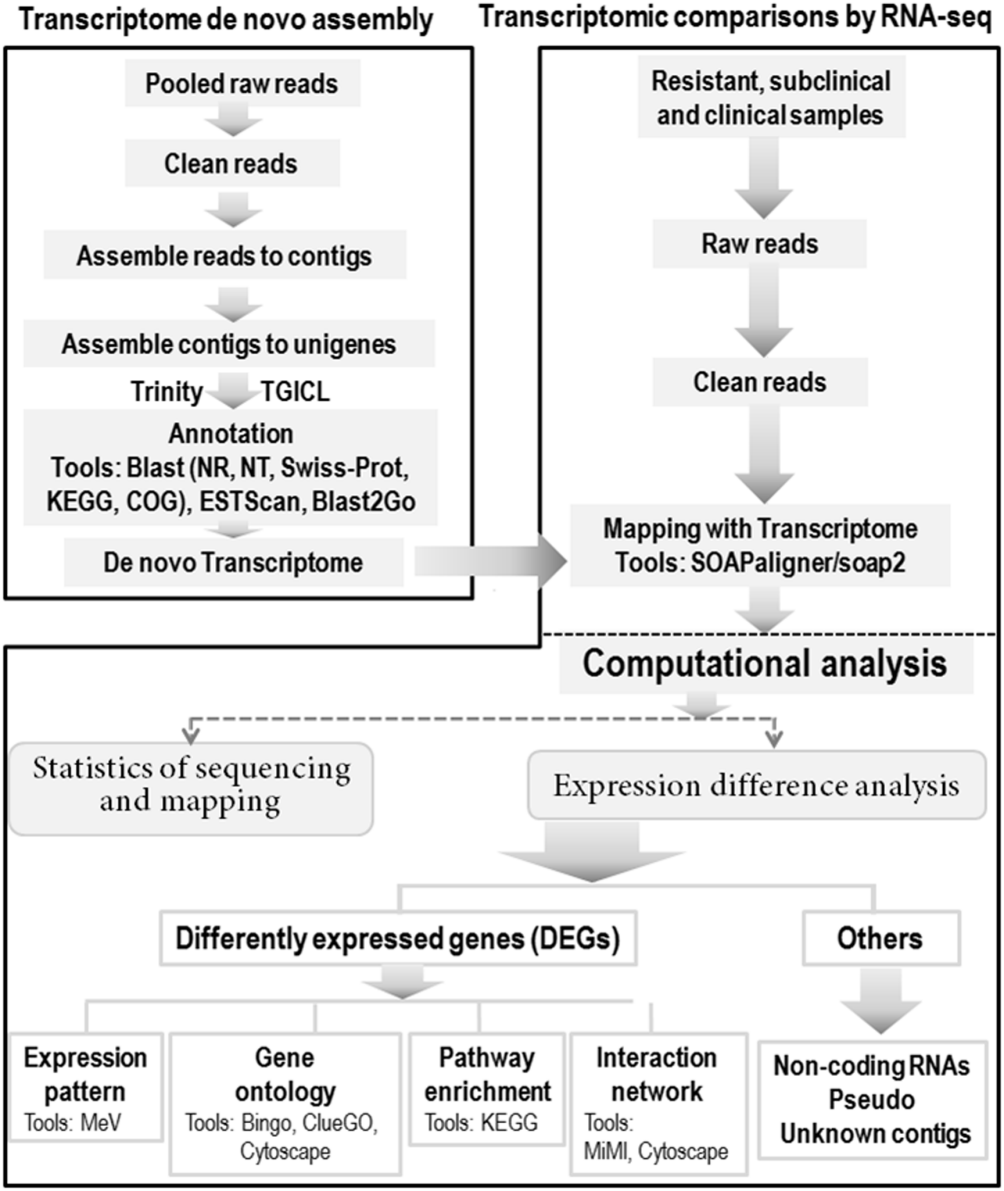
Bioinformatics workflows for data analysis. Transcriptome de(upper left), RNA-seqresult mapping and bioinformatic analysis of the transcriptomic sequences.

The library products were then subject to sequencing analysis via the Illumina sequencing platform (HiSeq 2000). The original image data generated by the sequence providers was transferred into nucleotide sequences data by base calling, defined as raw reads and saved as ‘fastq’ files. Raw reads produced from sequencing machines contain ‘dirty’ reads, from remaining trace adapters, and unknown or low quality bases, all of which could negatively affect bioinformatics analysis. Clean sequence reads were generated filtering the raw reads using three separate criteria, namely 1) removing reads with sequence adaptors, 2) removing reads in which unknown bases represent more than 10%, and 3) removing reads in which the percentage of low quality bases (quality value≤5) represents more than 50% in the read. All subsequent analyses were performed on these high-quality clean read data sets according to the bioinformatic analysis approach summarized in Fig. 1.

### De novo Transcriptome Assembly

The short read assembly program Trinity [17] was used to generate a sheep transcriptome de novo assembly. Briefly, the pair-end reads were combined according to length of overlap, to form contigs which were longer fragments matching the reads. Then the reads were mapped back to contigs to discriminate contigs from the same transcript, as well as the distances between these contigs, by using the information of pair-end reads. The Trinity tools then connected the contigs, generating sequences that cannot be extended on either and thus defining a set of Unigenes. Finally, the Unigenes were clustered using TGI Clustering tools (TGICL) [18] for obtaining the most distinct and longest non-redundant Unigenes possible. After clustering, the Unigenes were used for BLAST search and annotation against NCBI NR, NT and Swiss-Prot database, and the best alignments used to decide the sequence direction of the Unigenes. If a Unigene did not align with any of the above databases, ESTScan software [19] was used to decide sequence direction. The Blast2GO program [20] was used for initial functional annotation via gene ontology terms (GO; http://www.geneontology.org). Further annotation was then performed by BLASTing all Unigenes against Cluster of Orthologous Groups (COG) database and Kyoto Encyclopedia of Genes and Genomes (KEGG) database.

### Mapping of the Samples Reads to the Transcriptome and Analysis of Differentially Expressed Genes

Clean reads for resistant, subclinical and clinical experimental samples were mapped to the sheep reference transcriptome sequences using SOAPaligner/soap2 [21], allowing for no more than two 2 mismatched bases in the alignment for each read. Alignment statistical analysis was conducted to evaluate the sequencing and mapping quality, and to quantify the abundance of transcripts for each sample (Fig. S5–S6 in File S1). The level of gene expression was then calculated by the numbers of reads uniquely mapped to a reference sequences (Unigenes) using the RPKM [22] method (reads per kb per million reads) according to the formula below:
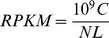
C = number of uniquely mappable reads that aligned to one UnigeneN = total number of uniquely mappable reads for all UnigenesL = number of bases on the Unigene

The coverage depth of each transcript was also determined (Fig. S7 in File S1). If there was more than one alternative transcript for a gene, the longest was used to calculate both its expression level and coverage. We then identified differentially expressed genes (DEGs) among resistant, subclinical and clinical samples [23] [24]. Fold changes for DEGs were calculated and FDR (false discovery rate) used to determine the threshold of P value for judging the significance of gene expression difference. The criterion for screening of differentially expressed genes was arbitrarily set as the following: FDR≤0.001 and fold change ≥2.

### Expression Pattern, Function Enrichment and Network Analysis of DEGs

**Figure 2:**
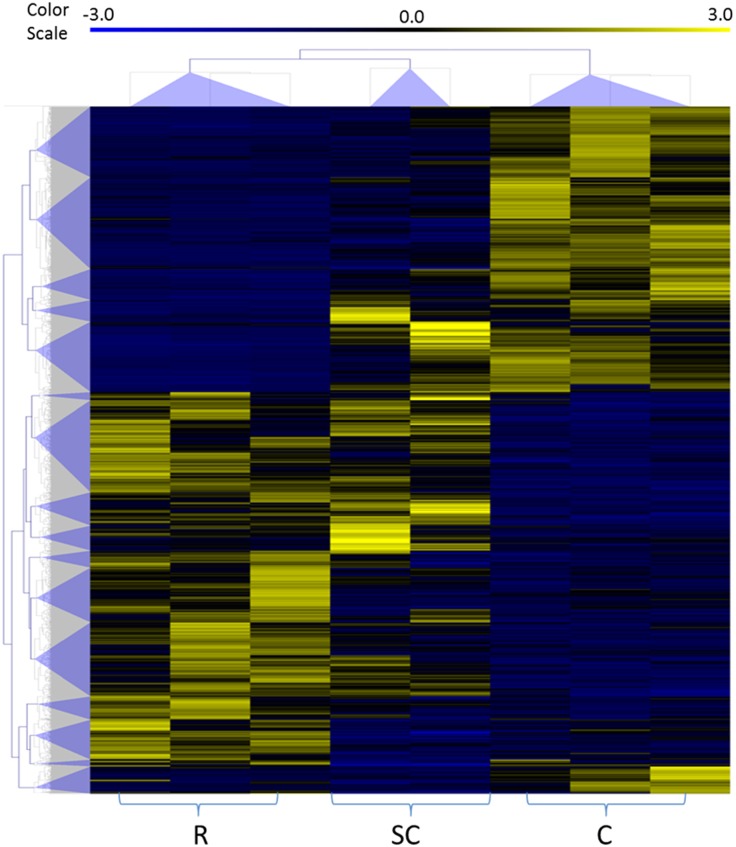
Hierarchical clustering. Hierarchical clustering of the 2414 contigs among 3 groups (from left to right: resistant, subclinical, clinical) using RPKM expression values from RNA-seq. We could identify 16 clusters vertically by visual inspection of the heat map.

Genes with similar expression patterns may be functionally and phenotypically correlated. We performed cluster analysis of gene expression patterns with TIGR MultiExperiment Viewer software version 4.9 [25] (Fig. 2). Each column represents an experimental sample (from resistant, subclinical and clinical group), while each row represents a DEG. Expression differences are shown in different colours with blue representing mean low levels of gene expression and yellow representing higher levels. To identify the principle biological functions of the DEGs we then mapped them to terms in KEGG database and GO database, looking for significantly enriched terms compared to the genomic background. DEGs were functionally grouped into gene ontology networks using the Cytoscape v2.8.3 software (http://cytoscape.org/index.php) with the ClueGO plug-in v1.8 (http://www.ici.upmc.fr/cluego/) [26]. ClueGO established a significant gene-term matrix and biologically functional groupings for all DEGs at different GO term levels. Pathway-based analysis further informed the biological functions of the DEGs, suggesting significantly enriched metabolic or signal transduction pathways associated to the DEGs, compared to the whole genomic background. The statistical significance of the terms analysed was calculated with two-sided enrichment/depletion hypergeometric test and Bonferroni P-value correction. In addition, we performed gene interaction network analysis by using Cytoscape plug-in MiMI [27] to connect to the MiMI database (http://mimi.ncibi.org) and view the involved interactions.

### Quantitative Real-Time PCR (qRT-PCR) Validation

A total of 1 ug DNase treated RNA was used for reverse transcription (RT), following the Superscript III (Invitrogen) manufacturer’s instructions. Samples were diluted 5 fold for Real Time PCR (RT-PCR) carried out on a Corbett Rotorgene 6000 (Qiagen) with SYBR ExTaq Mix (Takara), using multiple primers (Table 1) and a generic amplification sequence of 3 mins for initial denaturation, followed by 40 cycles at 95°C for 10 sec and 60°C for 25 sec. Transcripts were quantified relative to a geometric mean using three reference genes while normalising for different amplification efficiencies (denoted as “a”) as follows: expression level of gene of interest (GOI) = [aGOI∧(−CtGOI)]/([aREF1∧(−CtREF1)×aREF2∧(−CtREF2)×aREF3∧(−CtREF3)]∧(1/3)) [28]. Each sample was measured in triplicate.

**Table 1:** Primers used for qPCR analysis. REF:reference; GOI:gene of interest.

Gene	Forward	Reverse	Poppers
PPIA	GCATACAGGTCCTGGCATCT	TCTCCTGGGCTACAGAAGGA	REF
SDHA	GCAGAACCTGATGCTTTGTG	CGTAGGAGAGCGTGTGCTT	REF
YWHAZ	GCATCCCACAGACTATTTCC	GCAAAGACAATGACAGACCA	REF
GPX1	ACATTGAAACCCTGCTGTCC	TCATGAGGAGCTGTGGTCTG	GOI
SOD1	AGAGGCATGTTGGAGACCTG	CAGCGTTGCCAGTCTTTGTA	GOI
SOD2	AGCCATCAAACGTGAC	AGTGCCAACGATGACA	GOI
CAT	GATAATCGGGCCTGAC	CCCATGCTGCACATAG	GOI
CYP1A2	ACAACAAGGGATACAACAC	CGCTTGCGAACTTATCA	GOI
CYP2C8	TCACTGAGTTCCGTGCT	GGTGGTGTCGATGTCC	GOI
UGT2B10	TGGACGTGATTGGGTTT	GTCACAAGAGGATGGGAA	GOI
DKK3	GGGACCATCTGTGACAAT	GCTTACACACGTACACCA	GOI
SFRP1	AGTGCGACAAGTTTCC	AAAGGAAAACGGCGAC	GOI
WIF1	AGTGAACGTGATTGTCAT	ACCGGGAGTAACACAT	GOI
FABP5	AAGGCTTTGACGAATACAT	CATACCACCACTAATTTCCC	GOI
MMP14	CACTTTGACTCTGCCG	CTGGTAAAAGGGTGCC	GOI
ABCC1	GGATTTTTGCTATGGATCGT	GCACACAGTAGGGCTATAA	GOI
TOP2A	TATTCCGGTCCCGAAGA	CGCTTGTCATTCCGTT	GOI

## Results

### Sheep Trial

Facial Eczema (FE) mycotoxicity arises in grazing ruminants when the pasture contains toxic spores, and depending on temperatures and humidity, exposure may persist for a period of weeks or months [29]. In our study, liver samples were collected following a single exposure at seven weeks, empirically deemed a realistic time point to study the coping mechanisms of sheep. Based on the combination of blood GGT level and visible pathology, animals were grouped as ‘resistant’ (constant low GGT levels through the trial) and ‘susceptible’ (significantly increasing GGT levels), the latter group being further classified as ‘subclinical’ and ‘clinical’ on the basis of visually evident skin lesions(Table 2).

**Table 2:** Average GGT(U/L) through sheep trial.

Group	Week 0	Week 1	Week 2	Week 3	Week 4	Week 5
Resistant(n = 8)	35.6	49.4	49.9	44.4	43.8	44.5
Subclinical(n = 8)	56.0	92.8	828.8	1279.5	1286.9	1102.3
Clinical(n = 10)	44.1	96.5	972.6	1080.9	916.0	919.6

This table shows average blood GGT levels of resistant, subclinical and clinical sheep before treatment and first 5 weeks of trial.

In any clinical cases of FE, identified by skin lesions, are also manifest by seven weeks’ time. However, subclinical cases are not readily discernible, remaining cryptic to gross detection. In both clinical and sub clinical cases, liver damage is assumed to have begun earlier at around three weeks after exposure. GGT blood levels represent to first indicator exposure response [30], with a 20 fold increase in GGT level observed by the third week and persisting throughout the trial in susceptible individuals (Table 2). Skin lesions defining the clinical sub-group were not readily apparent at the does we used until late in the trial during the seventh week. Clinical phenotype individuals also showed a marked loss in body weight compared to the sub-clinical and resistant phenotype animals, which generally gained weight during the course of the trial (data not shown).

### De novo Reference Transcriptome Assembly and RNA-seq Transcripts Alignment

To date, the use of the sheep to investigate the genetic background for a certain disease model has been limited owing to the lack of transcriptome sequences and well-annotated ovine genome. Our purpose was therefore to generate an initial sheep transcriptome using next-generation technologies and to detect differentially expressed genes between resistant, subclinical and clinical sheep. We developed a bioinformatics pipeline for both de novo transcriptome assembly and the analysis of RNA-seq data based on this transcriptome (Fig. 1). A summary of the output of sequenced data, assembly quality and annotation results for the transcriptome work is presented in Table 3–5, including a total of 5.33 million clean reads with 4797.61 million clean nucleotides, a total of 84,243 Unigene with 71,302 distinct singletons for assembling. A total of 35,316, 62,824, and 32,892 Unigene was annotated to the public database NR, NT and SwissProt respectively. The clean reads for 8 experimental samples by RNA-seq were then mapped to the sheep reference, resulting in a high quality alignment. On average, about 78.5% of the short reads could be mapped (Table 6).

**Table 3:** The output of sequenced data indicates the completion of transcriptome work.

OutputStatistics	Total RawReads	Total CleanReads	Total CleanNucleotides(nt)	Q20percentage	Npercentage	GCpercentage
Reads	59,399,918	53,306,790	4,797,611,100	97.10%	0.00%	49.93%

Clean reads in each sample must contain a total base number of no less than the contractually required output. In this study, 2 reads are applied. Total Clean Nucleotides = Total Clean Reads1×Read1 size+Total Clean Reads2×Read2 size.

**Table 4:** Statistics of assembly quality is represented by number and length of contigs and unigenes.

Assembly quality	Total Number	Total Length(nt)	Mean Length(nt)	N50	Total Consensus Sequences	Distinct Clusters	Distinct Singletons
Contig	176,307	49,112,825	279	399	-	-	-
Unigene	84,243	48,519,596	576	967	84,243	12,941	71,302

The length distribution of the assembled sequences are showed in Fig. S1 in File S1.

**Table 5:** Unigenes were annotated with the databases of NR, NT, SwissProt, KEGG, COG and GO.

Annotation results	NR	NT	SwissProt	KEGG	COG	GO	ALL
Unigene	35,316	62,824	32,892	24,721	10,361	13,910	63,174

Then counted the number of unigenes annotated with each database. The summary annotation statistics for transcriptome are given in supplementary materials (Fig. S2, Fig. S3, and Fig. S4 in File S1) for NR classification, COG classification and GO classification respectively.

**Table 6:** RNA-seq alignment statistics.

Group	ID	Total Reads	Total BasePairs	Total Mapped Reads reads/percentage		Unique match reads/percentage	
Resistant	171	15273559	748404391	12406899	81.23%	9675136	63.35%
Resistant	199	15940439	781081511	12781923	80.19%	9862776	61.87%
Resistant	117	15437481	756436569	12484915	80.87%	9788271	63.41%
Subclinical	551	16467408	806902992	12850696	78.04%	10000198	60.73%
Subclinical	619	16179806	792810494	12708342	78.54%	9866337	60.98%
Clinical	184	16152120	791453880	12498828	77.38%	9690297	59.99%
Clinical	83	15191712	744393888	11549152	76.02%	8853099	58.28%
Clinical	36	15240757	746797093	11480775	75.33%	8847224	58.05%

This table discribes the read counts from RNA-seq and the rate of alignment to referent transcriptome for each individual sheep sample. The sequencing quality for each sample is summarized in Fig. S5 and Fig. S6 in File S1, including the classification of raw reads and the sequencing saturation analysis. The detailed alignment and annotatioon statistics are also showed in the supplementary materials Table S1 and Fig. S7 in File S1, representing the gene mapping and gene coverage information respectively.

### Clustering and Characteristic Expression Patterns

Hierarchical clustering was performed to identify individual animals with similar phenotypes as well as individual genes with similar expression profiles based on the RPKM expression values (Fig. 2). The heat plot displayed the expression patterns for all differentially expressed genes in all sheep. Horizontally, animals were identified as 3 clusters based on the expression patterns of all the annotated genes. The three derived clusters are completely aligned to the three defined phenotype outcomes observed after exposure. Vertically, genes identified from RNA-seq were separated into sixteen clusters based on their enrichment in each group.

### Expression Shift Profiles of Total and Detoxification Related Genes

**Figure 3:**
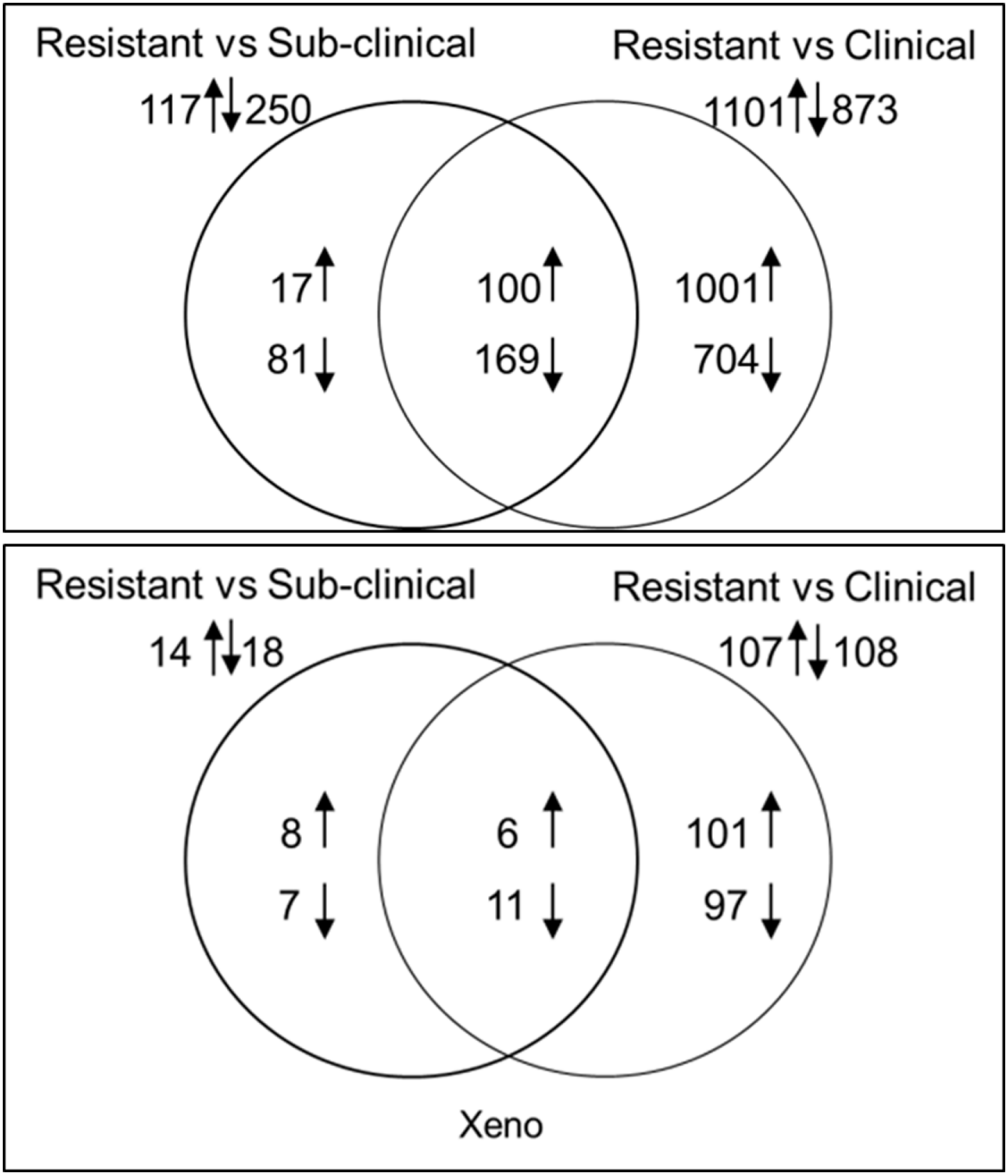
Vein diagrams of differently expressed genes among resistant, subclinical and clinical groups. The number of total differently expressed genes(upper) and detoxification related genes (lower) in two comparisons: resistant vs subclinical and resistant vs clinical. Number of genes that expressed higher or lower in resistant group was labeled with arrows.

Differential expression profile between sheep with different phenotypes was evaluated using RPKM method on the mapped reads as described above. There were a total of 2414 Unigenes differentially expressed between clinical, subclinical and resistant sheep. The basic types of aligned Unigenes are also listed in Table 7. By re-blasting all the differentially expressed Unigenes to public database NT, NR, and SwissProt especially for human, bovine, mouse, swine, and ovine, 1371, 1469, 1312, 1089 and 231 differentially expressed sequences are well aligned and annotated in the five species (Table 8). To identify the transcriptomic profile changes particularly in the terms of detoxification, we chose the genes that change between resistant and subclinical and genes that change between resistant and clinical, then separated genes under detoxification terms from the total differentially expressed genes (Fig. 3). The total number of genes that changed between resistant and subclinical is 367 with 117 expressed higher and 250 expressed lower in the resistant group. 1974 genes were changed between resistant and clinical with 1001 higher and 873 lower expressed in resistant group. There are 230 differentially expressed genes related to xenobiotic response process. 32 genes changed between resistant and subclinical group while 215 changes between resistant and clinical. Most of the expression changes happened between resistant and clinical groups rather than between resistant and subclinical groups.

**Table 7:** RNA type distribution of assembled sequences.

Type	Miscellaneous RNA	Protein coding	Pseudo	rRNA	Unknow	Total
Number	53	1379	39	1	908	2414

**Table 8:** Species distribution of annotated sequences.

Species	Human	Bovine	Mouse	Swine	Ovine	Total annotated
Number	1371	1469	1312	1089	231	1570

### Gene Ontology (GO) and Protein Interaction Analysis

**Figure 4:**
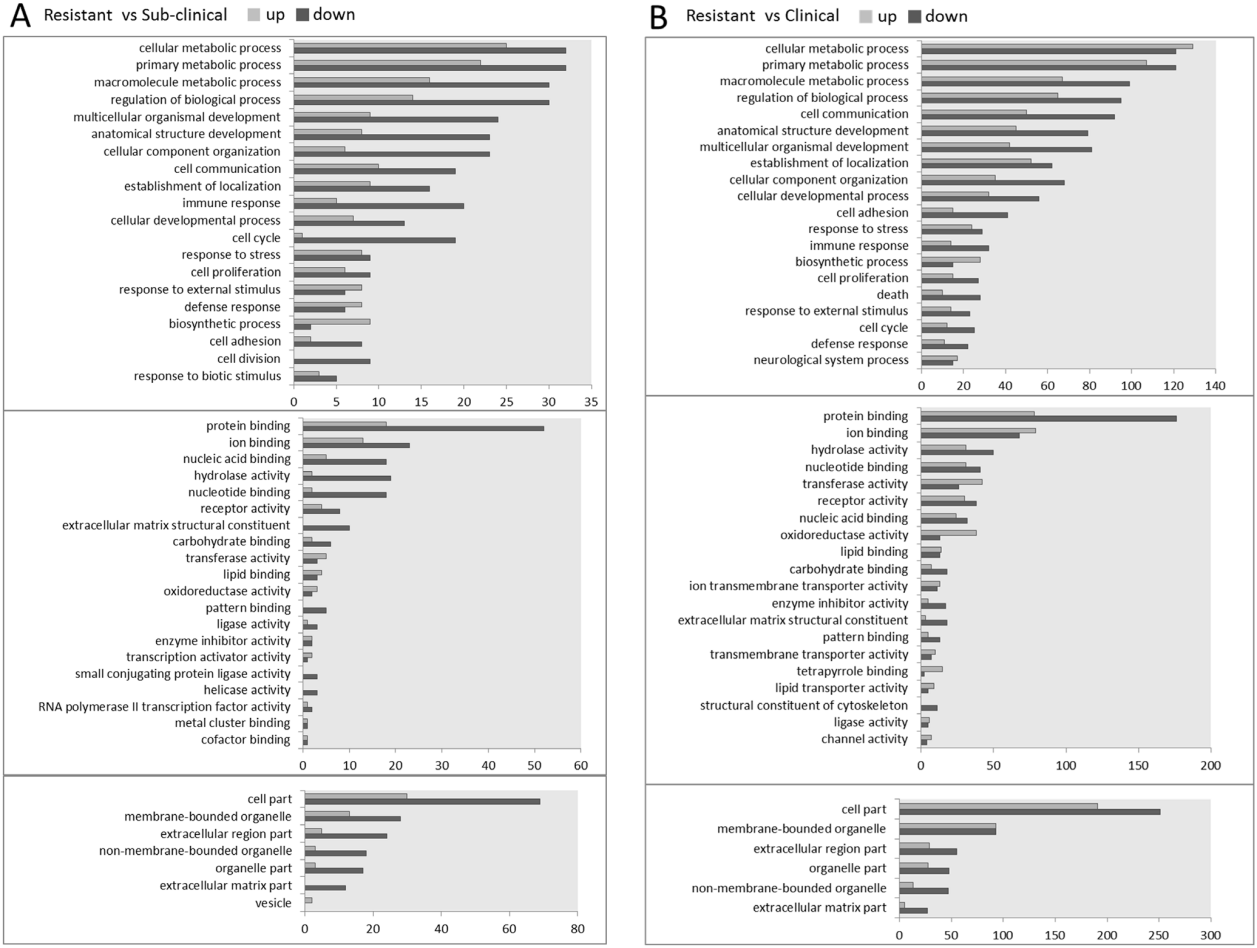
Top biological processes, molecular functions terms and cellular components that changed among groups. This figure describes the top 20 biological processes terms, top 20 molecular functions terms and top 6 cellular components that changed in two comparisons: A, resistant/subclinical and B, resistant/clinical from Go analysis.

Gene Ontology analysis including ‘biological processes’, ‘molecular functions’ and ‘cellular components’ was performed in all differentially expressed genes (Fig. S8–S10 in File S1). It was found the DEGs were significantly annotated to GO terms with well-known roles in oxidative stress and antioxidant protective processes, such as response to oxidative stress, omega-hydroxylase P450 pathway, epoxygenase P450 pathway, exogenous drug catabolic process, drug catabolic process, drug metabolic process, xenobiotic metabolic process, cellular response to xenobiotic stimulus, and response to xenobiotic stimulus. To specify each comparison, we further analysed both resistant/subclinical and resistant/clinical comparisons and listed top terms according to the number of annotated genes in Fig. 4. Predictably, both comparisons showed a shift in similar reference terms such as cellular metabolic process, cell communication, and response to stress. However, the number of involved genes that changed in the clinical sub-group is much greater than for the subclinical group, when compared to the resistant phenotype group.

### Pathway Analysis of Differentially Expressed Genes to Characterise the Transcriptomic Response of Sporidesmin Mycotoxin

**Figure 5:**
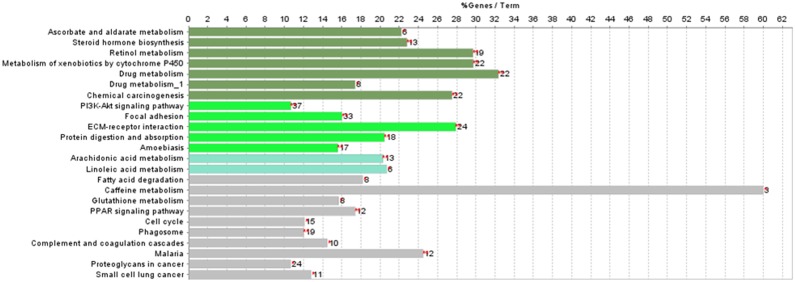
KEGG pathways and their function groups. The left lane shows the pathway name; the right lane shows the involved genes number (right side of each column chart) and their percentage for all the associated genes in each pathway or term (upper of the figure). The different colours for column charts represent different pathway functional grouping based on Kappa score.

For all the genes that differently expressed among groups, pathway enrichment analysis showed that a total number of 155 pathways were defined with at least one gene related to each pathway (File S2). To define the most significantly changed pathways, we primarily use the number of differentially expressed genes involved in each pathway as a standard. Focal adhesion, drug metabolism-cytochrome P450, and ECM-receptor interaction pathways are the three most significantly different biological roles thus identified. However, it is important to keep in mind that numbers of genes in each pathway are different. We additionally use the percentage change of known genes in each pathway as criterion. Caffeine metabolism, drug metabolism, and metabolism of xenobiotics by cytochrome P450 appear to be the top ones (Fig. 5). As for our study, the pathways of principle interest, namely detoxification and metabolism related pathways contain at least 4 differently expressed genes is listed in Table 9.

**Table 9:** Detoxification, metabolism related athways with at least 4 differently expressed genes and signaling related pathway with at least 4 differently expressed genes.

Pathways	Gene number	p-Value
**Detoxification related pathways**		
Drug metabolism - cytochrome P450	26	1.79E-30
Metabolism of xenobiotics by cytochrome P450	25	3.30E-29
Drug metabolism - other enzymes	11	6.62E-11
Porphyrin and chlorophyll metabolism	6	1.69E-05
ABC transporters - General	4	0.002822

### Quantitative RT-qPCR Validation of RNA-Seq Changes

**Figure 6:**
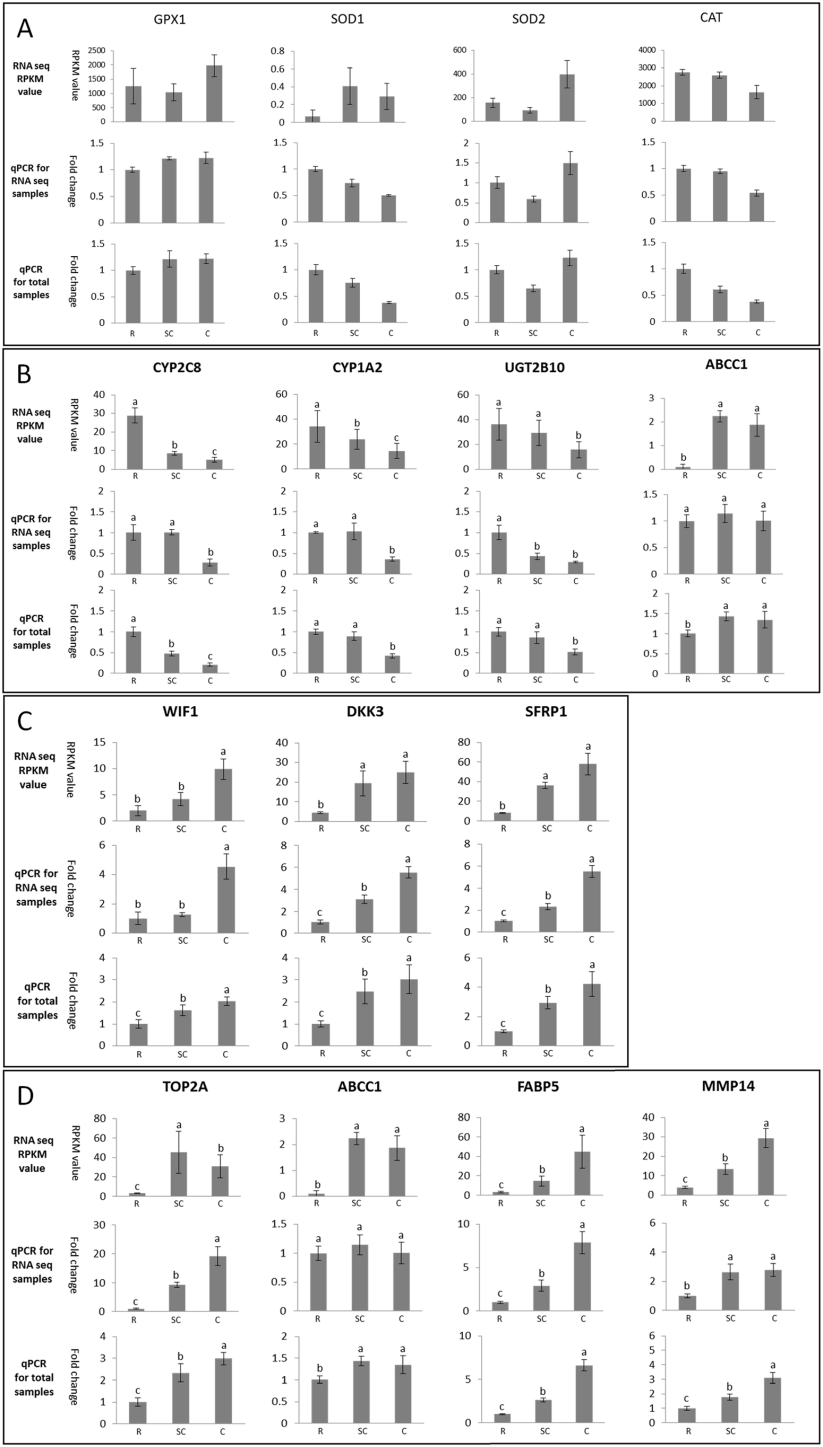
Quantitative RT-qPCR validation of RNA-Seq changes. For each gene, 3 comparisons are shown in the figure. Upper panel: fold changes between RNA-Seq RPKM value; middle panel: fold changes by qRT-PCR measured in the same subjects used for RNA-Seq analysis only; lower panel: fold changes by qRT-PCR in all subjects.

RT-qPCR of a subset of protein coding genes was done to confirm differential expression genes patterns derived from the discovery phase RNA-Seqn analysis. We provide a comparison of fold changes between RNA-Seq RPKM value and qRT-PCR for each gene for both the samples used only for RNA-Seq and across the entire trial population. The gene set includes antioxidant enzymes (Fig. 6-A), detoxification enzymes (Fig. 6-B), Wnt inhibitors (Fig. 6-C) and other randomly selected genes (Fig. 6-D). Virtually all the genes show concordant direction of fold change between RNA-Seq and qRT-PCR. The inconsistent seen with the SOD1 gene is likely due to a very low and incomplete RPKM value in RNA-seq.

### Statistical Significance of Results

Although increasingly affordable, whole genome analysis remains expensive in both assay cost and time for analysis by highly skilled practitioners. The expected richness of data offered by this approach has however encouraged its use, albeit on small cohort sizes. In some instances, triplicate sets have been found highly informative for sequencing analysis and have been widely used for RNA-Seq-related biological studies [31], although even a single sequencing run has been shown to be sufficient [32]–[34]. However, the importance of validating findings on biological replicates from the same populations cannot be understated and is an essential step in substantiating the readouts from RNA-seq analysis. In the current study, we found a high degree of consistency between the RNA-seq discovery platform and extension by qRT-PCR based to population wide validation. Critically, the fold changes in expression estimated from RNA-seq (n = 8) was highly correlated with that observed with qRT-PCR (n = 26) and the correlation coefficient reached 0.932993071 (p = 1.45948E-08), 0.783499202 (p = 0.000139968) and 0.454482048 (p = 0.064605227) for pairwise comparisons of NC vs C, R vs C and R vs NC respectively. We report these assessments of our replication success in support of the validity, accuracy and statistical power of our data despite the relatively small sample sizes being considered.

## Discussion

Mycotoxicity is frequently, but not invariably, the consequence of oxidative stress resulting from the generation of secondary metabolites after ingestion [5], [35]. It has long been appreciated that exposure to the mycotoxin sporidesmin in ruminants leads to tissue damage by free radicals [36] and there is a clear relationship between sporidesmin-induced liver injury and serum activity of gamma-glutamyltransferase in Romney lambs sired by both disease resistant and susceptible rams. However, we found that not all of the principal anti-oxidant enzymes were elevated as a correlate to disease progression, indeed catalase and GPX appearing to be refractory to toxin exposure. Furthermore, there was a selective response from the SOD family of enzymes, the expression of mitochondrial (type 2) and extracellular (type 3) SOD isoenzymes paralleling pathological progression, while the cytoplasmic (type 1) form of SOD was seen to be unchanged. While the antioxidant activities of SOD1 and the Se-dependent GPX1 are thought to be functionally coupled under some circumstances, dual genetic knock-out of these genes in mice does not lead to increased hepatic injury following endotoxin induced oxidative stress [37]. Our findings suggest that the functional coupling of SOD1 and GPX1 is similarly seemingly impartial to the effects of mycotoxin exposure.

The highly conserved nature of antioxidant enzymes across plant and animal taxa [38] has perhaps encouraged a tendency to view them as a functional collective. There is however a remarkable diversity in both the specific antioxidant mechanism and functional pathways which have evolved to cope, presumably in response to the manifold environmental triggers which are capable of disrupting cellular oxidative homeostasis, and a growing understanding of the specific contributions made by particular antioxidant enzyme in response to any given challenge. For example, the seasonal-long exposure of ruminants to a xenobiotic such as sporidesmin represents a persistent challenge. It has recently been demonstrated that chronic stress not only leads to a *differential* response by hepatic antioxidant enzymes, but also that the chronic stress response as a whole is a *modification* of, rather than distinct from, the response elicited by an acute stress stimulus [39].

Working in synergy with antioxidant mechanisms, a sophisticated network of multi-phase detoxification pathways has evolved to effectively neutralize and remove the toxic by-products of coping with free radicals and the derivative metabolites of the xenobiotic. A physiological level breed difference in detoxification of sporidesmin was first observed thirty years ago, with Merino sheep having a higher rate of metabolic turnover rate that Romney breeds [40]. In addition, using ‘sleep-time’ and recovery following sodium pentobarbital anaesthesia as an indicator of hepatic Phase I enzyme activity, Smith et al. again reported better detoxification of toxin in Merino than Romney breed rams. In these same animals, Merinos were found to be more tolerant of sporidesmin as indicated by the proxy measures of blood GGT, bodyweight change or photosensitisation [41]. We observed that several members of the cytochrome P450 (CYP) mono-oxygenase gene families 1–3, which are central to the Phase I biotransformation of xenobiotics [42], are differentially expressed between phenotypes following sporidesmin exposure. Unexpectedly, the highest expression of these genes was seen in those sheep exhibiting phenotypic ‘resistance’ to sporidesmin induced mycotoxicity. While the regulation of CYP genes is thought to occur at multiple levels, our knowledge of the particular mechanisms that lead to the induction of expression in particular instances is incomplete. Of the multiple CYPs we found to be elevated in the resistant phenotype, two (CYP1A2 and 2E1) are known to be subject to post-translational regulation and three (CYP1A2, 2B6 and 2C9) to transcriptional regulation [43]. The Ahr battery of transcriptional regulators has been linked to CYP1A2, while xenobiotic nuclear receptors, such as the constitutive androstane receptor (CAR) and pregnane X receptor (PXR) which function as ligand induced transcription factors to enhance biotransformation [44], have been shown to induce CYP2B6 (CAR) and 2C9 (CAR and PXR) [45]. Additionally, the beta-catenin intracellular signalling pathway has been shown to regulate the expression of multiple CYP genes, using *in vitro* human HepG2 hepatocytes [46], murine in vivo expression [47] [48], and perhaps most significantly to modulate *in vivo* the extent to which murine hepatocyte CYP genes respond to xenobiotic challenges [49]. Beta-catenin is the central element of, and stabilized by the canonical Wnt pathway leading to transcriptional activation of genes by the transcription factor complex Tcf/LEF. Canonical signalling by Wnt1 and 3a in particular, impacts on liver physiology (particularly metabolism) and pathology [50]. In prostate cancers, canonical Wnt signalling has been found to activate Ahr activity [51] and may act as a co-stimulator of Ahr target gens [49]. Meanwhile, the binding of non-canonical forms of Wnt (such as Wnt4 and 5a) leads to other distinct intracellular signalling pathways.

A shear panoply of extracellular and intracellular factors are known to either positively or negatively regulate both canonical and non-canonical Wnt signalling pathways, in both non-mammalian and mammalian development, during tissue homeostasis and in human diseases [52]. These include two classes of secreted extracellular anatgonists which interfere with ligand-receptor binding [53]. Members of the secreted Frizzled-receptor protein (SFRP) family, Wnt inhibitory factor (WIF)-1 and cerberus molecules bind directly to Wnt proteins in order to block binding to the Wnt receptor complex and can inhibit both canonical and non-canonical signalling. Meanwhile, members of the Dikkopf (DKK) family of molecules antagonize by binding specifically to components of the Wnt receptor complex and inhibit canonical signalling. Strikingly, we found differential transcriptional suppression of three key major Wnt antagonists, sRFP1, WIF-1 and Dkk3, suggesting that multiple Wnt signalling pathways, and in particular the amplification of CYP gene dependent detoxification, may be occurring in hepatic tissue of sheep exhibiting a sporidesmin ‘resistant’ phenotype. Of the multiple isoenzymes found to be more highly expressed in these sheep, CYP1A2 and CYP2E1 are both reliant on beta-catenin dependent transcription [47], [48].

In Phase II of metabolic detoxification, the original xenobiotic compound or the intermediate metabolites modified during Phase I are conjugated in preparation for excretion via the biliary duct. Multiple families of enzymes, including glutathione S transferases (GSTs) and UDP glycurosyltranferases (UGTs), contribute to Phase II processing. In parallel with the CYP genes, we found several hepatic GST and UGT family members to be differentially expressed and correlated to phenotype following sporidesmin exposure. Furthermore, for at least some of those which are elevated after sporidesmin exposure there is already evidence that beta-catenin is important in their regulation, genetic ablation of the beta-catenin encoding gene Ctnnb1 in human and murine leading to a loss of GSTM1, GSTA3 and GSTA4 expression [49], [50] while an activating mutation in beta-catenin in hepatocellular cancers leading to increased expression of GSTM and GSTA4 [54].

The novel findings presented here suggest that potentiation of Wnt dependent detoxification mechanisms may be a characteristic feature that defines a favourable outcome following sporidesmin challenge in ruminants, and significantly alters the perception that effective treatment of mycotoxicoses needs always to be predicated on a need to reverse oxidative stress induced damage. Rather, enhancing detoxification may provide a more successful pathway for remediation. Our findings suggest a new perspective for potential diagnosis and treatment of mycotoxicoses in ruminants by identifying novel molecular pathways which would seem to regulate the extent of hepatic response to xenobiotic challenge.

### Availability of Supporting Data

The sequencing data from the experiments involved in this work can be accessed from the Short Read Archive at the National Centre for Biotechnology Information (http://www.ncbi.nlm.nih.gov/sra) under the accession number SRA102080, and the specifically NCBI accession numbers for each raw data are SRX359140, SRX359141, SRX359259, SRX359297, SRX359298, SRX359299, SRX359300, SRX359301 (project submission ID: SRP030219).

## Supporting Information

File S1 Figure S1. The length distribution of the assembled sequences. Figure S2. NR classification of blast alignment for the assembled sequences. Figure S3. Cluster of Orthologous Groups (COG) for the assembled sequences. Figure S4. Gene Ontology (GO) analysis for the assembled sequences. Figure S5. The classification of raw reads for resistant, subclinical and clinical experimental samples. Figure S6. The sequencing saturation for resistant, subclinical and clinical experimental samples. Figure S7. The distribution of mapping genes’ coverage for resistant, subclinical and clinical experimental samples. Figure S8. Gene Ontology analysis result of cellular components term. Figure S9. Gene Ontology analysis result of biological processes term. Figure S10. Gene Ontology analysis result of molecular functions term. Table S1. Alignment Statistics for RNA-seq reads mapping.
(PDF)

File S2 List of KEGG Pathway analysis results.
(XLSX)
