# Supplementary material for: RNA-Seq Analysis Implicates Detoxification Pathways in Ovine Mycotoxin Resistance
Source: PLoS One. 2014 Jun 17;9(6):e99975. doi: 10.1371/journal.pone.0099975 (PMC4061066; doi:10.1371/journal.pone.0099975)
Supplement: File S1 — Figure S1. The length distribution of the assembled sequences. Figure S2. NR classification of blast alignment for the assembled sequences. Figure S3. Cluster of Orthologous Groups (COG) for the assembled sequences. Figure S4. Gene Ontology (GO) analysis for the assembled sequences. Figure S5. The classification of raw reads for resistant, subclinical and clinical experimental samples. Figure S6. The sequencing saturation for resistant, subclinical and clinical experimental samples. Figure S7. The distribution of mapping genes’ coverage for resistant, subclinical and clinical experimental samples. Figure S8. Gene Ontology analysis result of cellular components term. Figure S9. Gene Ontology analysis result of biological processes term. Figure S10. Gene Ontology analysis result of molecular functions term. Table S1. Alignment Statistics for RNA-seq reads mapping. (PDF) [file pone.0099975.s001.pdf]

Supplementary figures

1. Transcriptome assembly

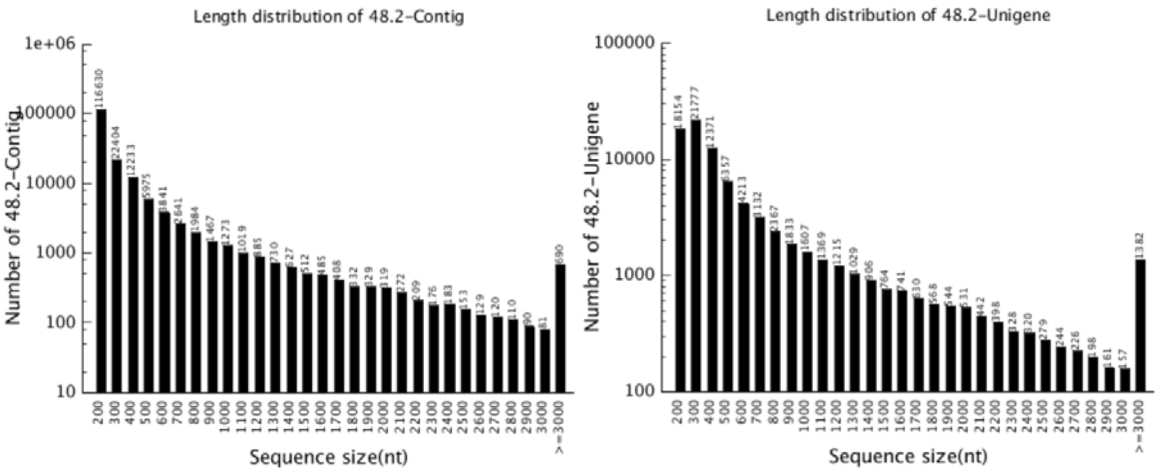

FigureS1. The length distribution of the assembled sequences

2. Transcriptome annotation

1). NR classification for the transcriptome

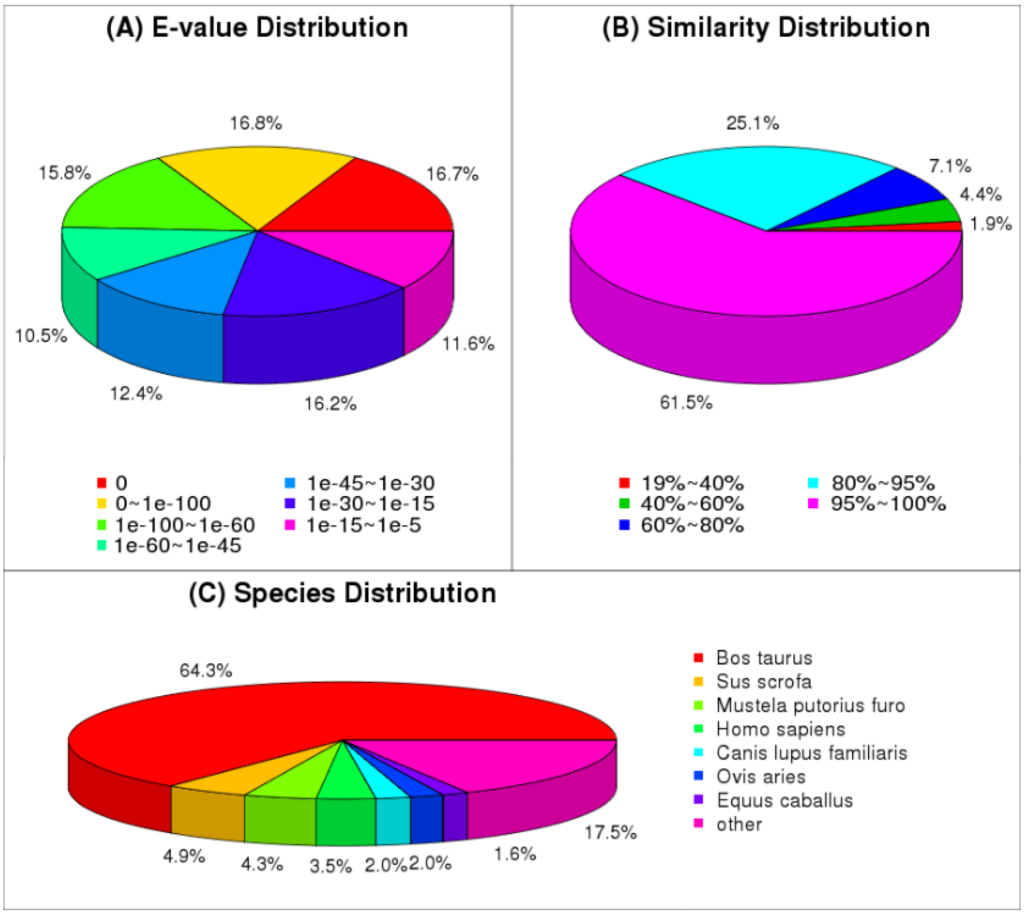

FigureS2. NR classification of blast alignment for the assembled sequences

Notes:

(A) Figure of E-value distribution; (B) Figure of identity distribution; (C) Figure of species distribution

2). COG classification for the transcriptome

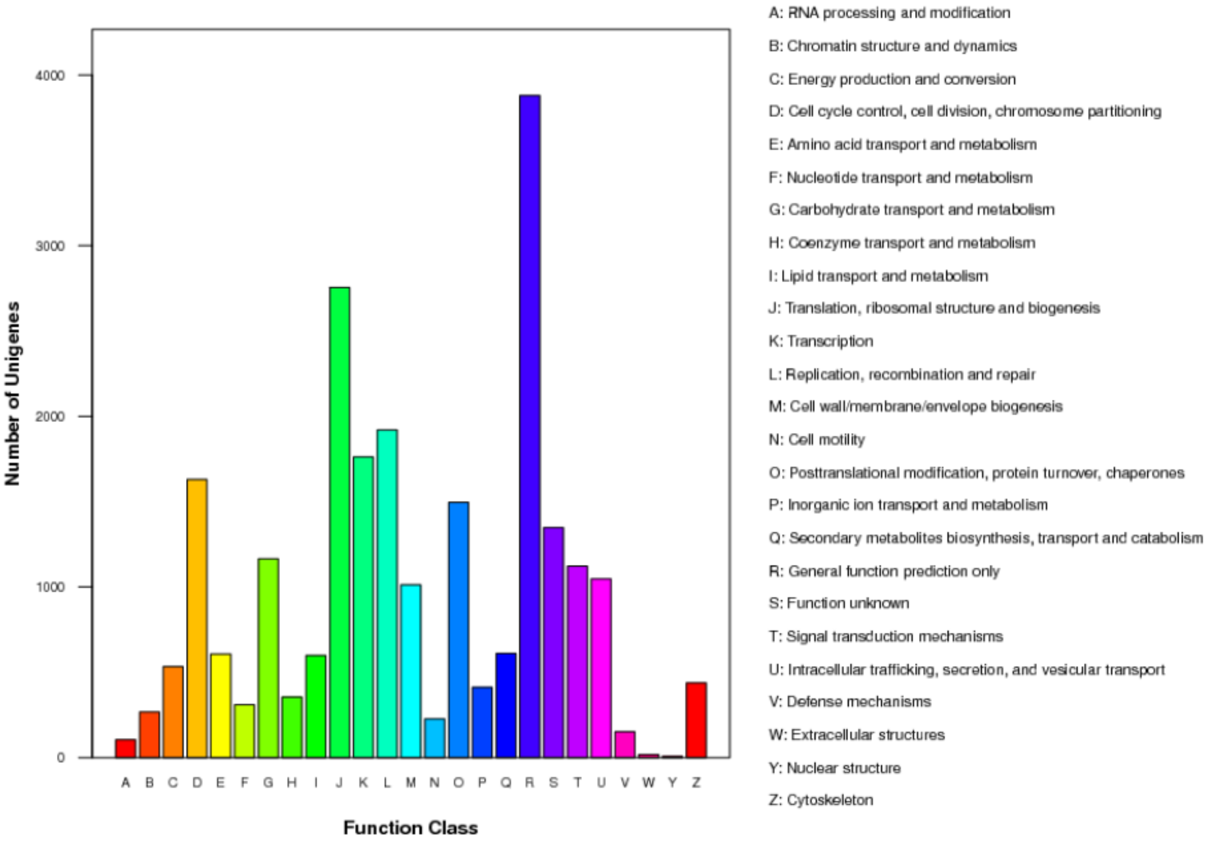

FigureS3. Cluster of Orthologous Groups (COG) for the assembled sequences

3). GO classification for the transcriptome

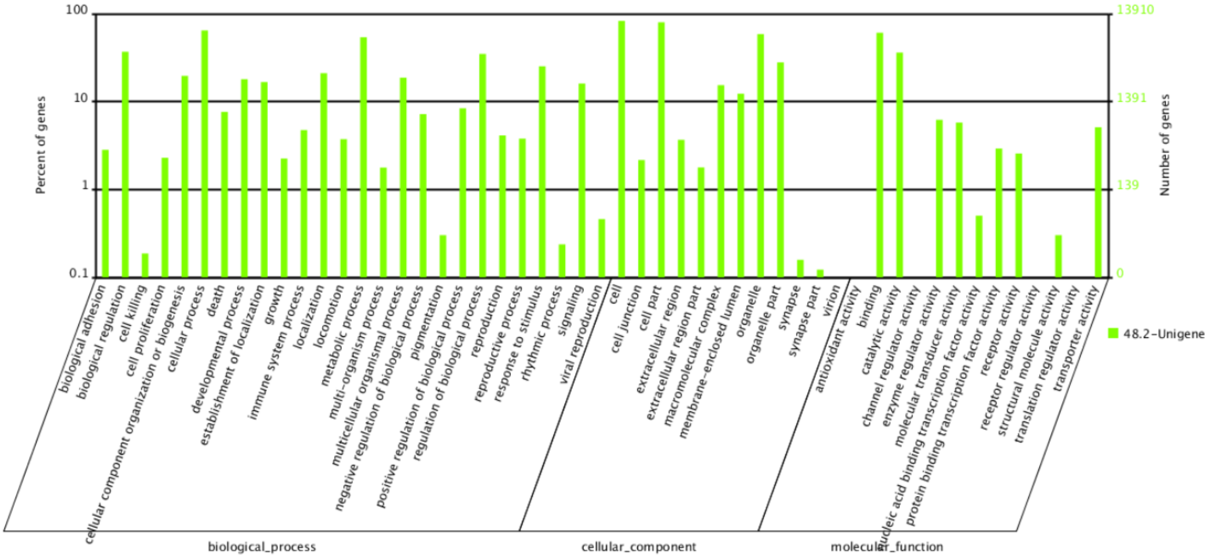

FigureS4. Gene Ontology (GO) analysis for the assembled sequences

3. Transcriptomic comparisons

1). Sequencing Quality Evaluation

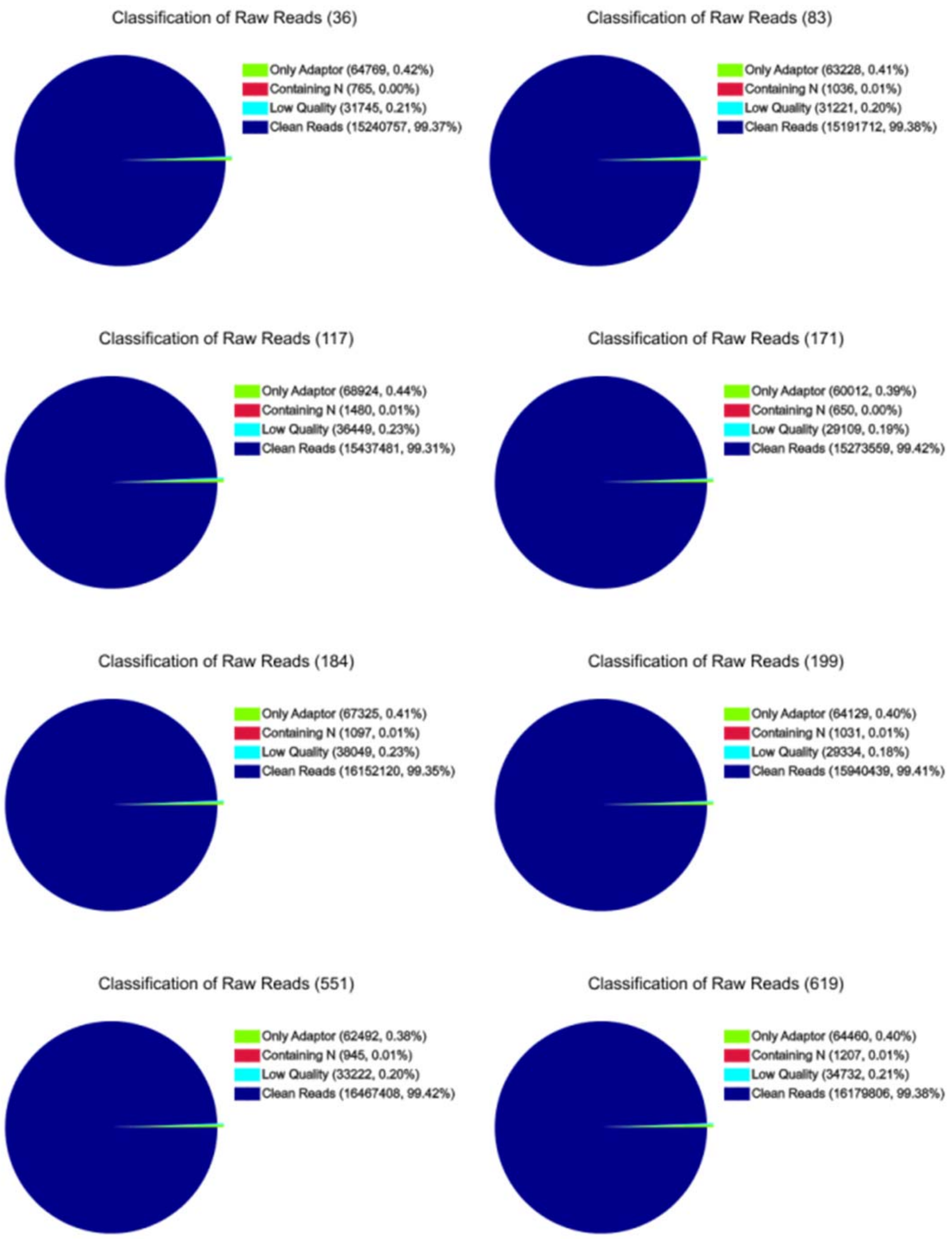

FigureS5. The classification of raw reads for resistant, subclinical and clinical experimental samples.

2). Sequencing Saturation Analysis

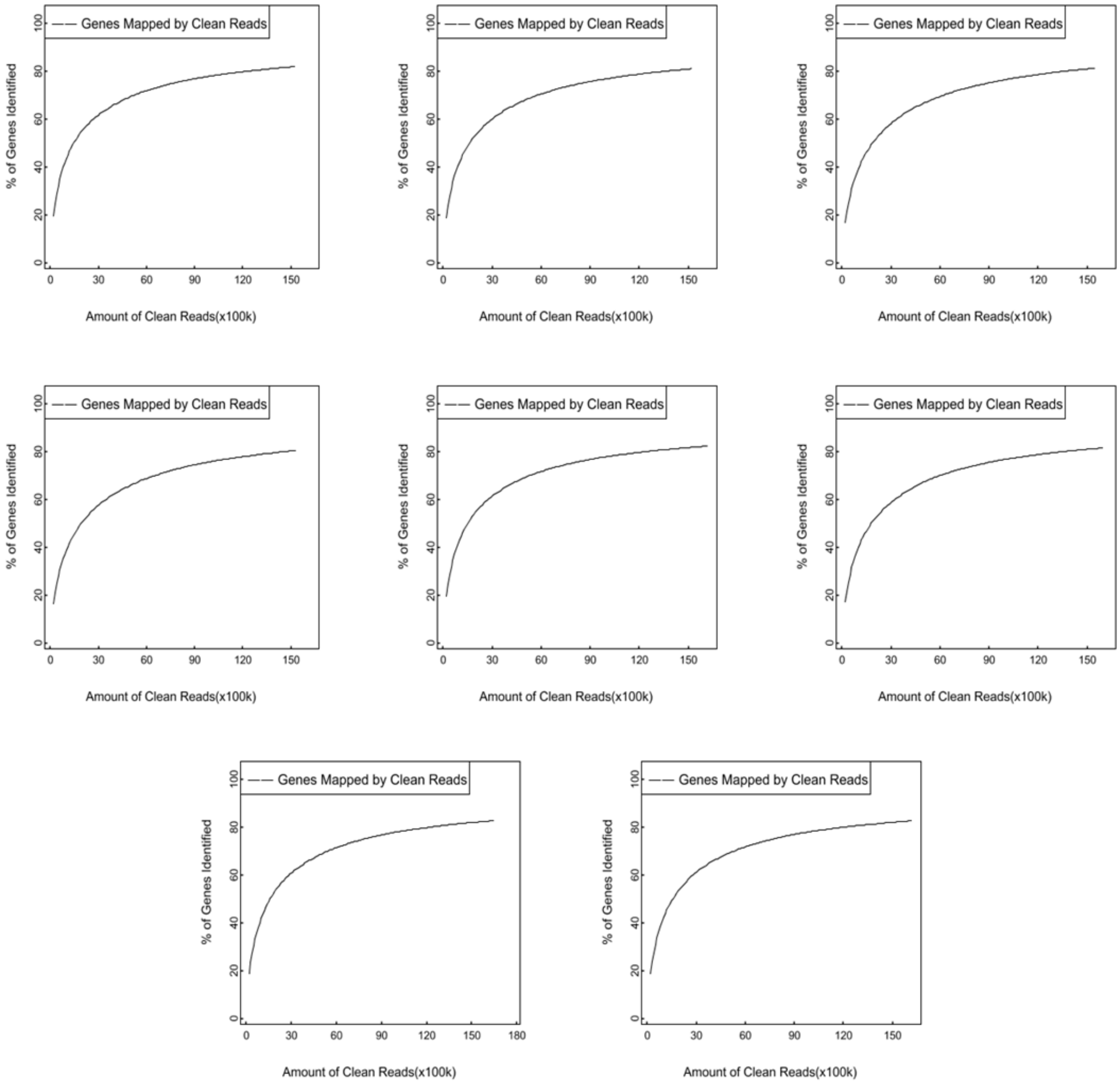

FigureS6. The sequencing saturation for resistant, subclinical and clinical experimental samples.

Note: the first row represented 36, 83, 117 respectively; the second row 171, 184, 199; and the third row 551, 619 respectively.

3). Alignment Statistics for mapping to transcriptome

TableS1. Alignment Statistics for RNA-seq reads mapping.

117: Stat. of Map to Gene

Map to Gene	reads number	percentage
Total Reads	15437481	100.00%
Total BasePairs	756436569	100.00%
Total Mapped Reads	12484915	80.87%
perfect match	8299358	53.76%
<=2bp mismatch	4185557	27.11%
unique match	9788271	63.41%
multi-position match	2696644	17.47%
Total Unmapped Reads	2952566	19.13%

171: Stat. of Map to Gene

Map to Gene	reads number	percentage
Total Reads	15273559	100.00%
Total BasePairs	748404391	100.00%
Total Mapped Reads	12406899	81.23%
perfect match	8257748	54.07%
<=2bp mismatch	4149151	27.17%
unique match	9675136	63.35%
multi-position match	2731763	17.89%
Total Unmapped Reads	2866660	18.77%

184: Stat. of Map to Gene

Map to Gene	reads number	percentage
Total Reads	16152120	100.00%
Total BasePairs	791453880	100.00%
Total Mapped Reads	12498828	77.38%
perfect match	8394403	51.97%
<=2bp mismatch	4104425	25.41%
unique match	9690297	59.99%
multi-position match	2808531	17.39%
Total Unmapped Reads	3653292	22.62%

199: Stat. of Map to Gene

Map to Gene	reads number	percentage
Total Reads	15940439	100.00%
Total BasePairs	781081511	100.00%
Total Mapped Reads	12781923	80.19%
perfect match	8944338	56.11%
<=2bp mismatch	3837585	24.07%
unique match	9862776	61.87%
multi-position match	2919147	18.31%
Total Unmapped Reads	3158516	19.81%

36: Stat. of Map to Gene

Map to Gene	reads number	percentage
Total Reads	15240757	100.00%
Total BasePairs	746797093	100.00%
Total Mapped Reads	11480775	75.33%
perfect match	7606280	49.91%
<=2bp mismatch	3874495	25.42%
unique match	8847224	58.05%
multi-position match	2633551	17.28%
Total Unmapped Reads	3759982	24.67%

551: Stat. of Map to Gene

Map to Gene	reads number	percentage
Total Reads	16467408	100.00%
Total BasePairs	806902992	100.00%
Total Mapped Reads	12850696	78.04%
perfect match	8929282	54.22%
<=2bp mismatch	3921414	23.81%
unique match	10000198	60.73%
multi-position match	2850498	17.31%
Total Unmapped Reads	3616712	21.96%

619: Stat. of Map to Gene

Map to Gene	reads number	percentage
Total Reads	16179806	100.00%
Total BasePairs	792810494	100.00%
Total Mapped Reads	12708342	78.54%
perfect match	8484007	52.44%
<=2bp mismatch	4224335	26.11%
unique match	9866337	60.98%
multi-position match	2842005	17.57%
Total Unmapped Reads	3471464	21.46%

83: Stat. of Map to Gene

Map to Gene	reads number	percentage
Total Reads	15191712	100.00%
Total BasePairs	744393888	100.00%
Total Mapped Reads	11549152	76.02%
perfect match	7728224	50.87%
<=2bp mismatch	3820928	25.15%
unique match	8853099	58.28%
multi-position match	2696053	17.75%
Total Unmapped Reads	3642560	23.98%

4). Gene coverage statistics

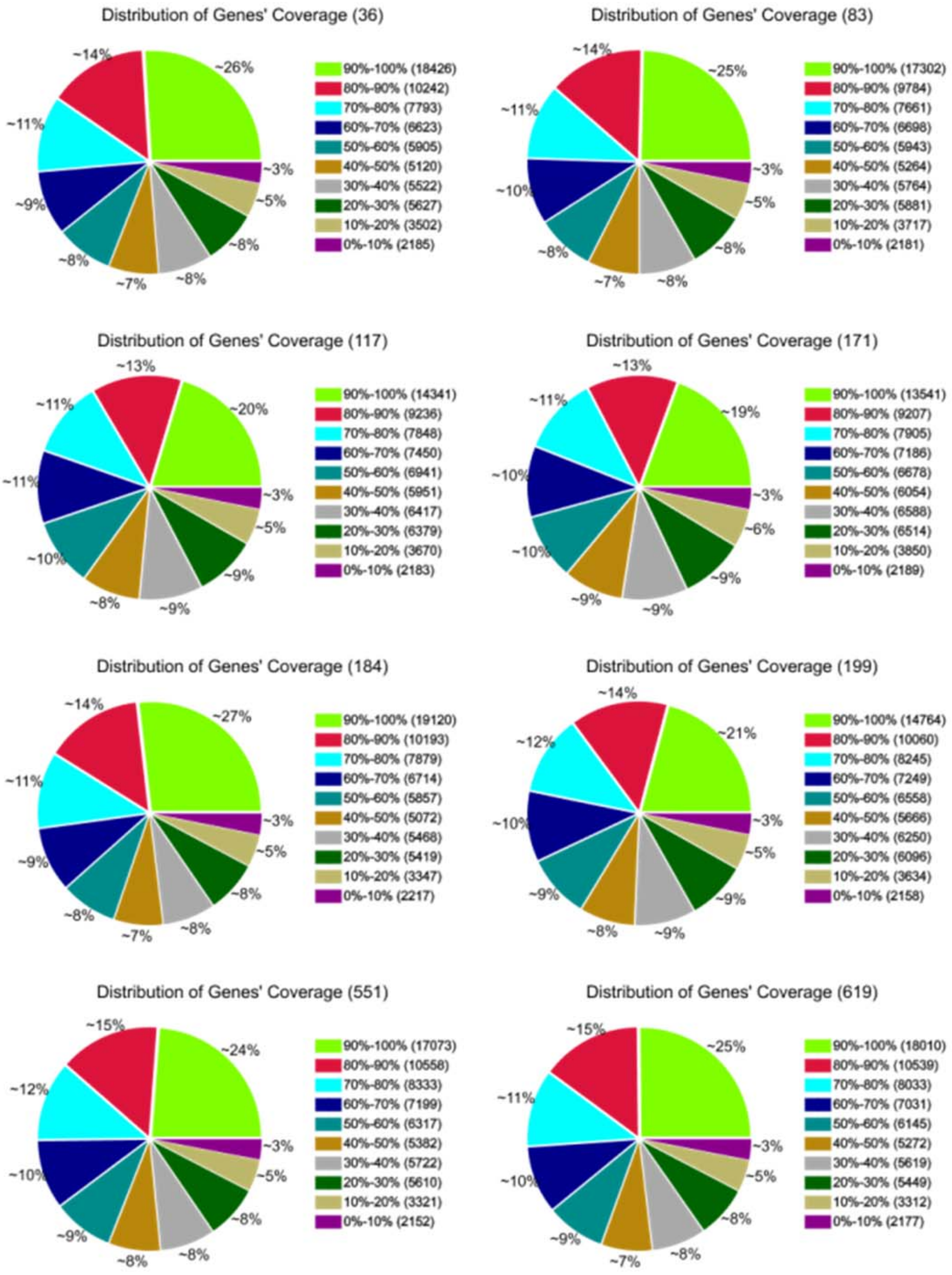

FigureS7. The distribution of mapping genes' coverage for resistant, subclinical and clinical experimental samples.

4. Go analysis

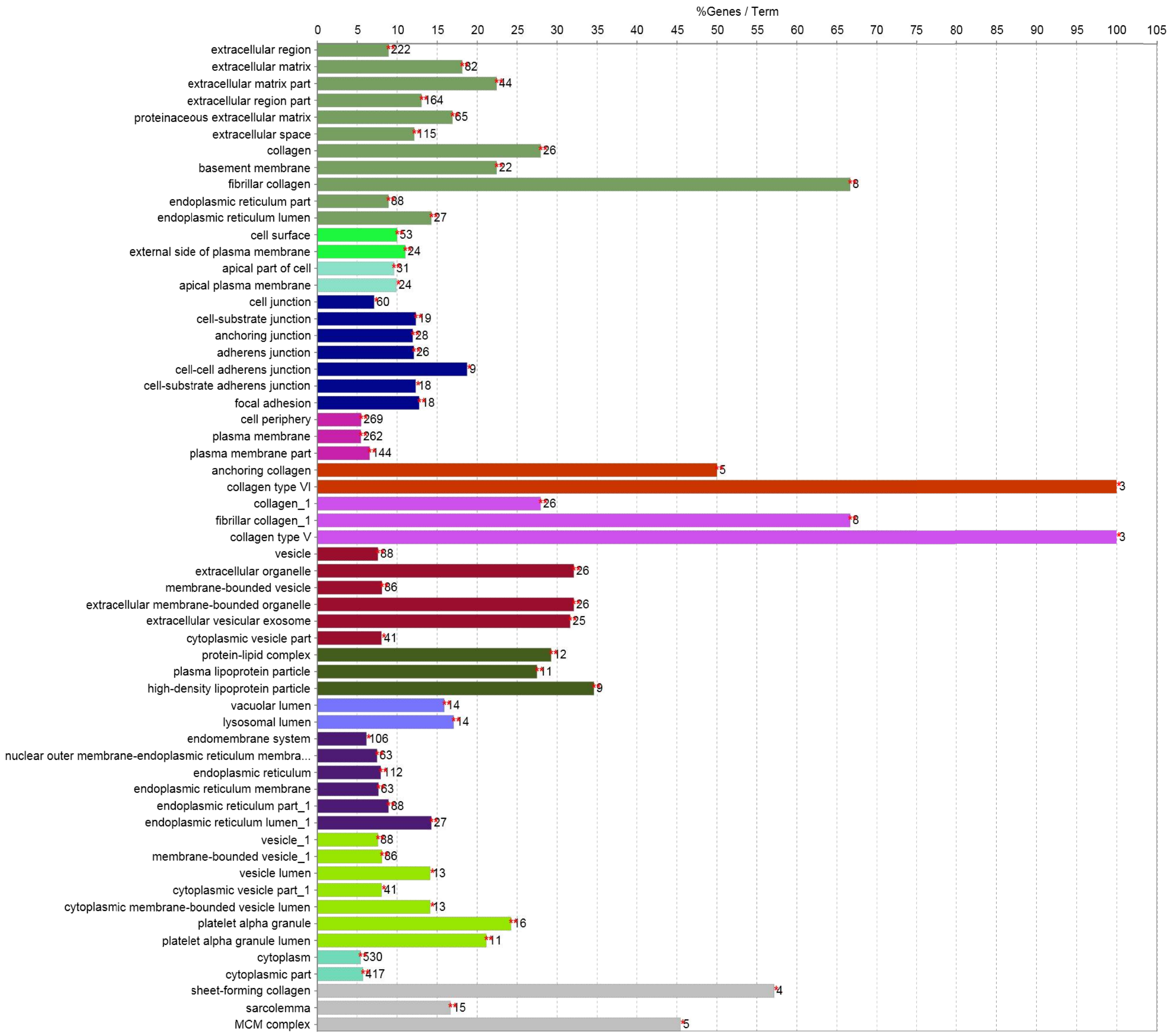

FigureS8. Gene Ontology analysis result of cellular components term

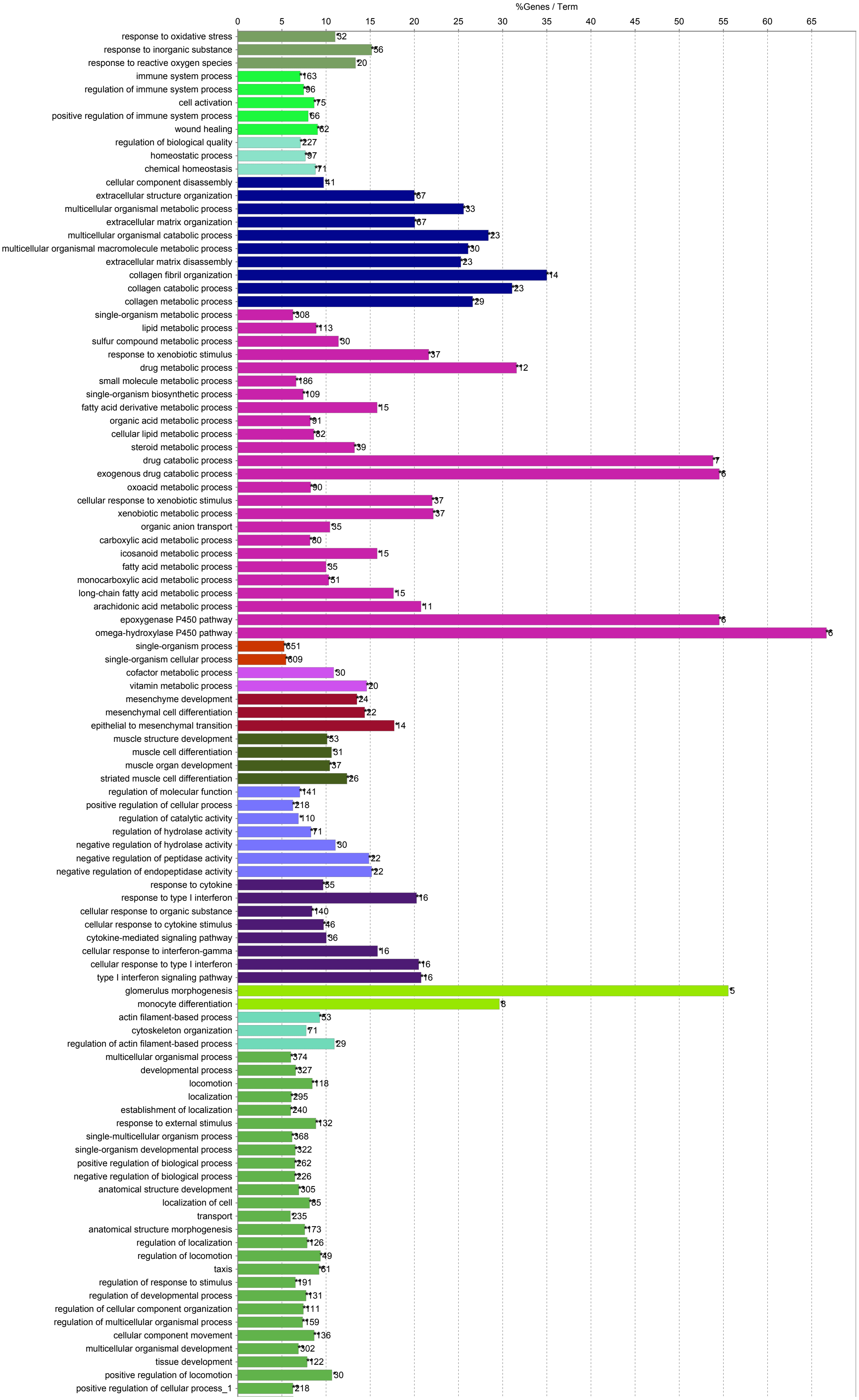
%Genes / Term
0 5 10 15 20 25 30 35 40 45 50 55 60 65
response to oxidative stress 32
response to inorganic substance 56
response to reactive oxygen species 20
immune system process 163
regulation of immune system process 96
cell activation 75
positive regulation of immune system process 66
wound healing 62
regulation of biological quality 227
homeostatic process 97
chemical homeostasis 71
cellular component disassembly 41
extracellular structure organization 67
multicellular organismal metabolic process 33
extracellular matrix organization 67
multicellular organismal catabolic process 23
multicellular organismal macromolecule metabolic process 30
extracellular matrix disassembly 23
collagen fibril organization 14
collagen catabolic process 23
collagen metabolic process 29
single-organism metabolic process 308
lipid metabolic process 113
sulfur compound metabolic process 30
response to xenobiotic stimulus 37
drug metabolic process 12
small molecule metabolic process 186
single-organism biosynthetic process 109
fatty acid derivative metabolic process 15
organic acid metabolic process 91
cellular lipid metabolic process 82
steroid metabolic process 39
drug catabolic process 7
exogenous drug catabolic process 6
oxoacid metabolic process 90
cellular response to xenobiotic stimulus 37
xenobiotic metabolic process 37
organic anion transport 35
carboxylic acid metabolic process 80
icosanoid metabolic process 15
fatty acid metabolic process 35
monocarboxylic acid metabolic process 51
long-chain fatty acid metabolic process 15
arachidonic acid metabolic process 11
epoxygenase P450 pathway 6
omega-hydroxylase P450 pathway 6
single-organism process 651
single-organism cellular process 609
cofactor metabolic process 30
vitamin metabolic process 20
mesenchyme development 24
mesenchymal cell differentiation 22
epithelial to mesenchymal transition 14
muscle structure development 53
muscle cell differentiation 31
muscle organ development 37
striated muscle cell differentiation 26
regulation of molecular function 141
positive regulation of cellular process 218
regulation of catalytic activity 110
regulation of hydrolase activity 71
negative regulation of hydrolase activity 30
negative regulation of peptidase activity 22
negative regulation of endopeptidase activity 22
response to cytokine 55
response to type I interferon 16
cellular response to organic substance 140
cellular response to cytokine stimulus 46
cytokine-mediated signaling pathway 36
cellular response to interferon-gamma 16
cellular response to type I interferon 16
type I interferon signaling pathway 16
glomerulus morphogenesis 5
monocyte differentiation 8
actin filament-based process 53
cytoskeleton organization 71
regulation of actin filament-based process 29
multicellular organismal process 374
developmental process 327
locomotion 118
localization 295
establishment of localization 240
response to external stimulus 132
single-multicellular organism process 368
single-organism developmental process 322
positive regulation of biological process 262
negative regulation of biological process 226
anatomical structure development 305
localization of cell 85
transport 235
anatomical structure morphogenesis 173
regulation of localization 126
regulation of locomotion 49
taxis 61
regulation of response to stimulus 191
regulation of developmental process 131
regulation of cellular component organization 111
regulation of multicellular organismal process 159
cellular component movement 136
multicellular organismal development 302
tissue development 122
positive regulation of locomotion 30
positive regulation of cellular process_1 218

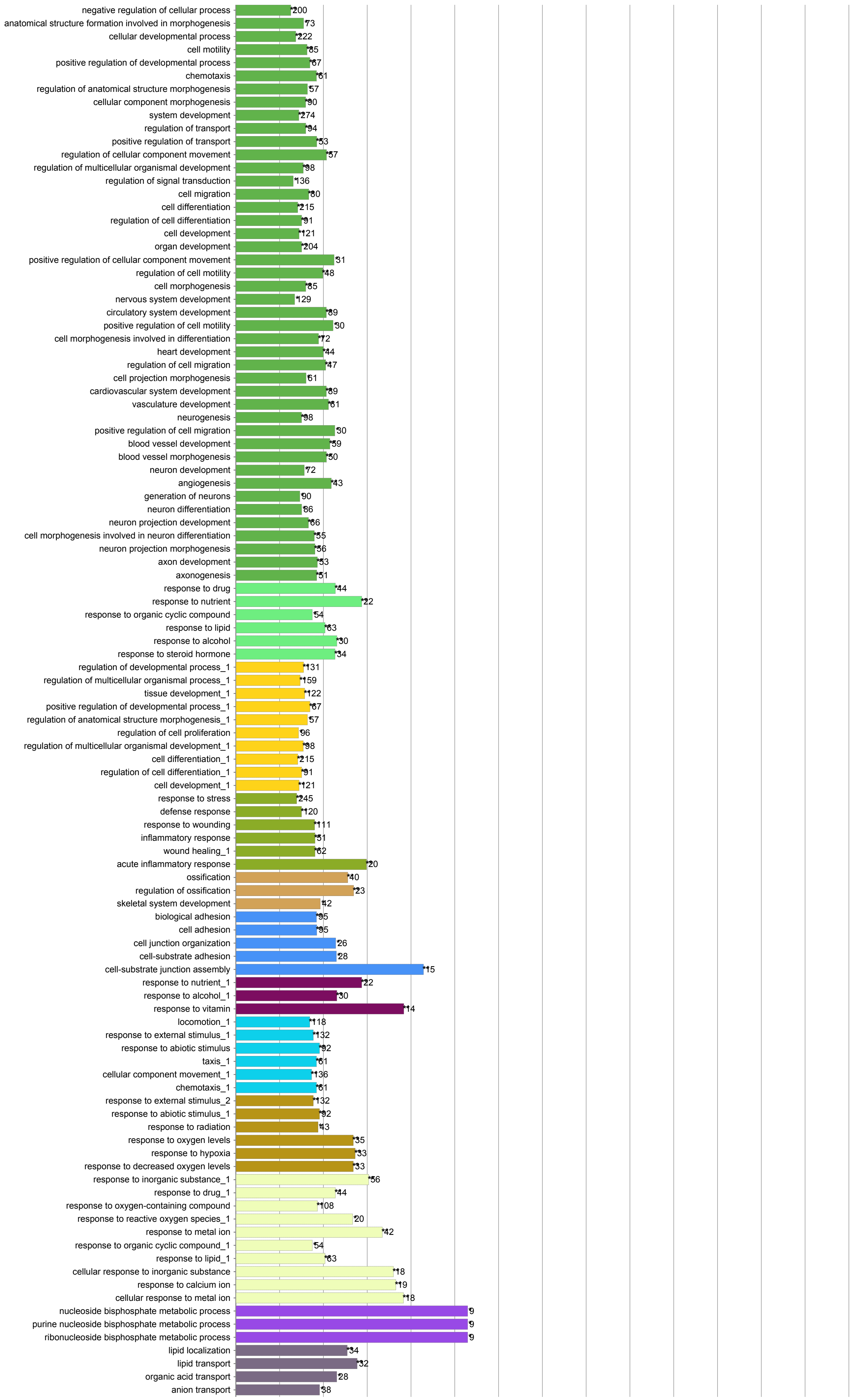

negative regulation of cellular process *200
anatomical structure formation involved in morphogenesis *73
cellular developmental process *222
cell motility *85
positive regulation of developmental process *67
chemotaxis *61
regulation of anatomical structure morphogenesis *57
cellular component morphogenesis *90
system development *274
regulation of transport *94
positive regulation of transport *53
regulation of cellular component movement *57
regulation of multicellular organismal development *98
regulation of signal transduction *136
cell migration *80
cell differentiation *215
regulation of cell differentiation *91
cell development *121
organ development *204
positive regulation of cellular component movement *31
regulation of cell motility *48
cell morphogenesis *85
nervous system development *129
circulatory system development *89
positive regulation of cell motility *30
cell morphogenesis involved in differentiation *72
heart development *44
regulation of cell migration *47
cell projection morphogenesis *61
cardiovascular system development *89
vasculature development *61
neurogenesis *98
positive regulation of cell migration *30
blood vessel development *59
blood vessel morphogenesis *50
neuron development *72
angiogenesis *43
generation of neurons *90
neuron differentiation *86
neuron projection development *66
cell morphogenesis involved in neuron differentiation *55
neuron projection morphogenesis *56
axon development *53
axonogenesis *51
response to drug *44
response to nutrient *22
response to organic cyclic compound *54
response to lipid *63
response to alcohol *30
response to steroid hormone *34
regulation of developmental process_1 *131
regulation of multicellular organismal process_1 *159
tissue development_1 *122
positive regulation of developmental process_1 *67
regulation of anatomical structure morphogenesis_1 *57
regulation of cell proliferation *96
regulation of multicellular organismal development_1 *98
cell differentiation_1 *215
regulation of cell differentiation_1 *91
cell development_1 *121
response to stress *245
defense response *120
response to wounding *111
inflammatory response *51
wound healing_1 *62
acute inflammatory response *20
ossification *40
regulation of ossification *23
skeletal system development *42
biological adhesion *95
cell adhesion *95
cell junction organization *26
cell-substrate adhesion *28
cell-substrate junction assembly *15
response to nutrient_1 *22
response to alcohol_1 *30
response to vitamin *14
locomotion_1 *118
response to external stimulus_1 *132
response to abiotic stimulus *92
taxis_1 *61
cellular component movement_1 *136
chemotaxis_1 *61
response to external stimulus_2 *132
response to abiotic stimulus_1 *92
response to radiation *43
response to oxygen levels *35
response to hypoxia *33
response to decreased oxygen levels *33
response to inorganic substance_1 *56
response to drug_1 *44
response to oxygen-containing compound *108
response to reactive oxygen species_1 *20
response to metal ion *42
response to organic cyclic compound_1 *54
response to lipid_1 *63
cellular response to inorganic substance *18
response to calcium ion *19
cellular response to metal ion *18
nucleoside bisphosphate metabolic process *9
purine nucleoside bisphosphate metabolic process *9
ribonucleoside bisphosphate metabolic process *9
lipid localization *34
lipid transport *32
organic acid transport *28
anion transport *38

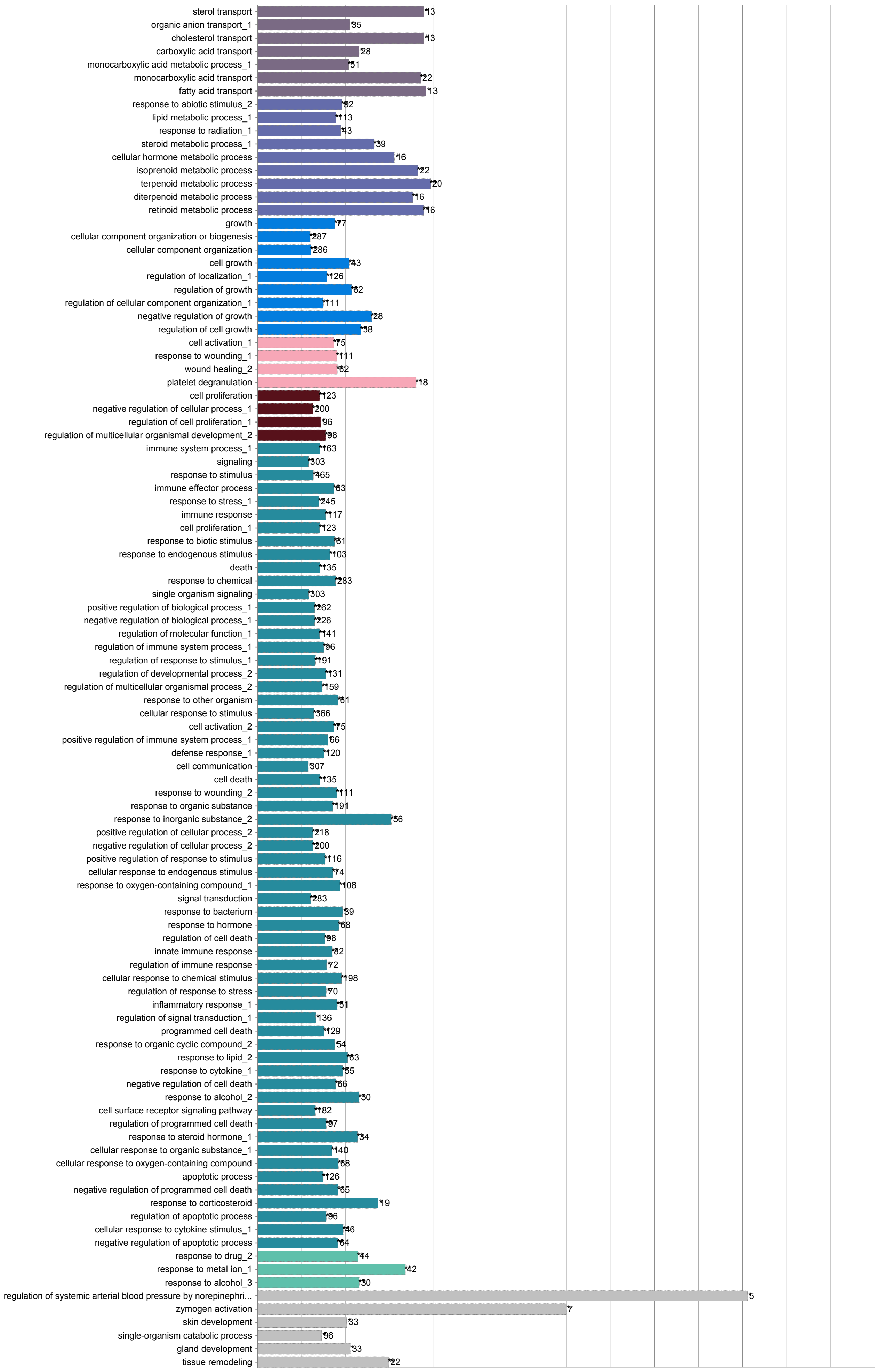

FigureS9. Gene Ontology analysis result of biological processes term

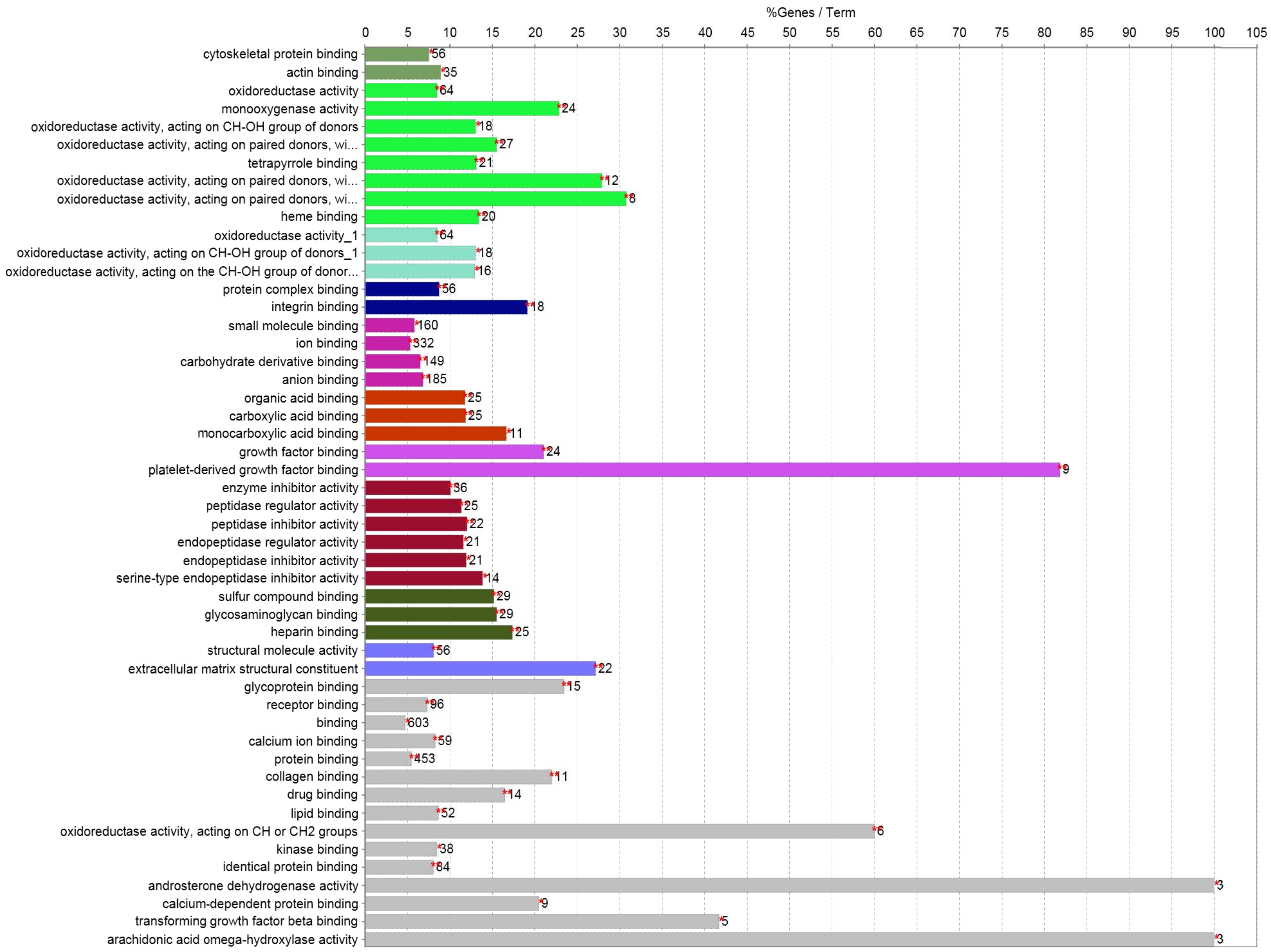

FigureS10. Gene Ontology analysis result of molecular functions term
